# Social construction of the image of the psychologist and of the patient: the role of implicit premises

**DOI:** 10.3389/fpsyg.2023.1233091

**Published:** 2023-08-25

**Authors:** Josephine Convertini, Margherita Luciani

**Affiliations:** ^1^Department of Psychiatry, Lausanne University Hospital (CHUV), Lausanne, Switzerland; ^2^Usi in ascolto Service, Università della Svizzera italiana (USI), Lugano, Switzerland

**Keywords:** psychologist, patient, contextual premises, framing, stigma

## Abstract

Nowadays, there is a growing consideration of people's mental health through awareness programs, policies, and practices promoted by international aid agencies and non-governmental organizations. Psychologists and patients are major actors in mental health, and their images are socially co-constructed. Nevertheless, there is still a lot of confusion about who “psychologists” and “patients” are or what a psychologist does. This muddle may underline stereotypes and broadly speaking stigma related to mental health. Therefore, confronting directly the ideas of “psychologist” and “patient” could be a little step in challenging stereotypes and making order in the panorama of mental health. In our study, we focus on the implicit contextual premises that shape particular framings around which the images of the psychologist and of the patient are socially and culturally co-constructed. In order to reach this goal, we have investigated the discourses and the multiple points of view behind the social image of the psychologist and of the patient from different sources or contextual domains: psychology online forums, university websites, and an online survey. From a methodological perspective and according to the pragma-dialectical approach, we have identified all the different standpoints and arguments related to the various conceptions of the psychologist and the patient. We have made explicit the implicit premises that lay behind each argumentative inference via the Argumentum Model of Topics. Based on these analyses, we have reconstructed the distinct framings at stake in the different contextual domains. The findings show that implicit contextual premises have huge power in constructing stigmatization in the ideas that lay people have toward the image of the psychologist and of the patient. In particular, we have observed that the more the contextual domain is defined, the more institutional premises dominate over individual ones; on the contrary, in informal contextual domains, heterogenous individual premises are prominent. Our study underlines that it is only by substituting old implicit premises with new unimagined ones that we can change subjacent contextual premises at the very core of stigma and the prototypical world's images.

## 1. Introduction

The role of the psychologist can be defined according to different criteria. Following the European Certificate in Psychology (EuroPsy), “the overall purpose of practicing as a professional psychologist is to develop and apply psychological principles, knowledge, models and methods in an ethical and scientific way in order to promote the development, well-being and effectiveness of individuals, groups, organizations and society” (p. 45, 2021).^1^ Furthermore, according to the Italian law (No. 56/89), “the profession of psychologist includes the use of cognitive and intervention tools for prevention, diagnosis, habilitation-rehabilitation and support activities in the field of psychology addressed to the person, group, social bodies and communities. It also includes experimentation, research and teaching activities in this field”.^2^ In addition to this, psychologists have to carry out many other ethical, moral, and deontological duties. The APA Dictionary of Psychology defines a psychologist as “an individual who is professionally trained in one or more branches or subfields of psychology (…). Psychologists work in a variety of settings (...). The professional activities of psychologists are also varied but can include psychological counseling, involvement in other mental health care services, educational testing and assessment, research, teaching, and business and organizational consulting”.^3^ Several differences in psychologist, psychotherapist, and counseling training practices still remain worldwide. For example, discrepancies concern whether or not attending personal therapy sessions during the training (Edwards, 2018), the quality standards of psychotherapy supervision practices (Watkins, 2013), and the guidance regarding trainees' evaluation (Fiorillo et al., 2011). Despite some misalignments that can be found in the training, a widely shared agreement concerns the presence of professional criteria regulating the profession of psychology.

Referring to the person who goes to the therapy,^4^ several labels such as patient, client, and user can be found according to the reference documents (e.g., DSM-5, psychological associations' status, and event flyers). However, these terms are not exhaustive of all the characteristics entailed by a person who decides to consult a psychologist. Indeed, lay people develop a diversity of images of the “patient”: who they are and the reasons behind consulting a psychologist. There is still a lot of confusion around the social images of the psychologist and the patient.

Moreover, in the panorama of mental health, both the psychologist and the patient can be the object of stereotypes, prejudices, and discriminations (e.g., ageism refers to stereotypes toward others or oneself based on age, stereotypical, or inequitable gender attitudes). Social stigma is a widely studied phenomenon. Nevertheless, as WHO recently reported^5^ (WHO, 2022), many steps in the context of mental health are still to be pursued. Our study can be situated in line with these studies (e.g., Heijnders and Van Der Meij, 2006; Orchowski et al., 2006) and, by directly confronting the ideas of “psychologist” and “patient”, we aim to offer a little contribution in challenging stereotypes and in making order in the panorama of mental health. In particular, our study deals with the investigation of implicit premises leading to the stereotypes that rotate around the figures of the psychologist and of the person who consults a psychologist.

In Section 2, we present a theoretical framework proposing the notion of “perfect ideas” and a brief literature review on stigmatizing frameworks in psychology. Section 3 is devoted to the methodology adopted to collect and analyze the data. Section 4 presents the findings by illustrating four excerpts in a detailed way. In Section 5, we conclude the study.

## 2. Theoretical framework

We stage our research in the context of the literature on stigma in psychology. Moreover, we adopt the concept of “perfect ideas” (Cecchin and Apolloni, 2003) to present the stigma as the result of rigid ideas perpetrated in cultural premises.

### 2.1. The tyranny of the totalitarian glance of “perfect ideas”

In this study, we follow the path traced by Cecchin and Apolloni (2003), which coin the concept of “perfect ideas”, i.e., “results of pragmatic actions and behaviors, aimed at managing the present time, that as a result of their evident utility and efficacy tend to be structured in terms of absolute, a-temporal and a-historical truth”^6^ (Cecchin and Apolloni, 2003, p. 44). This is similar to what is called “musts” in cognitive psychology: irrational and rigid beliefs on the image that people have on themselves, on the others, and on the life conditions (Ellis, 1989), as for example: “If I am not able to find a job, I am an unworthy person”. These beliefs are absolutistic and dogmatic and are acquired by human beings in the form of rules eliciting emotional distress (Ellis and Harper, 1975). Moreover, they are conceived as implicit or explicit premises that guide people in their interactions with the world and with the social environment and that lead to irrational conclusions. Nevertheless, perfect ideas are much more than simple musts: Indeed, they are ideas that include not only logical but also contextual premises.

It is the normative idea entailed in the logical underpinnings of Cecchin's perfect ideas that lead people to blind labyrinths (Cecchin and Apolloni, 2003). What are perfect ideas according to Cecchin? In short, they are ideas that people have on the way in which the world *should* be and on how people themselves *should* be: In particular, these representations are perceived as obligations with reference to what the familiar, relational, and socio-cultural premises “prescribe”. It is like a Totalitarianism and an authoritarian glance that people impose themselves alone. We are dealing with social worlds created in light of practices oriented toward *stasis* and perfection, which create pathological systems and stigmas. This way of living and interacting with the external world and with one's internal world is pervasive and gives rise to what Cecchin has called “stuck systems”, i.e., immobile systems (Cecchin and Apolloni, 2003) in which the same immobile relational dynamics are perpetrated. Perfect ideas, which sustain and are at the same time sustained by stuck systems, have one main protagonist: freezing premises in an immutable condition without any possibility of change and without the possibility to imagine a new state of affairs.

As highlighted by Bateson (1976), our way of knowing reality is characterized by an alternation of descriptions of “processes” and classifications of “forms”: When we perceive simple actions, gestures, and behaviors, we immediately classify them into categories of actions. For instance, if we see a person taking food from another, we can start to classify it into categories of actions (buying, selling, donating, and stealing). This happens because we have a totalitarian glance toward phenomena and we make inferences on the basis of our premises (Cecchin and Apolloni, 2003). This highly resembles what Ferreira has called “familiar myths”, i.e., rigid ideas consisting of shared and well-rooted thoughts that refer to each member of a given family and the reciprocal positions within familiar life. These thoughts are not challenged by anyone in the family, even though they are characterized by big reality's distortions (Ferreira, 1963): Myths constrain subsequent generations to repeat the same schemes, therefore occupying the positions that maintain a balance in a given family.

As psychologists, we are continuously faced with rigid categories that identify the way in which we connect ourselves with the world. This conception fits very well with the study on stigma that we have conducted, since we believe that stigma has to do with rigid ideas perpetrated in cultural premises over time, and which become obvious ahistorical premises.

### 2.2. Stigma in psychology

Stigma is a phenomenon broadly explored in the literature on psychotherapy, and it is associated with having mental illness or mental disorders, seeking psychotherapy (Lannin et al., 2013), maintaining relationships with people having psychological problems (Feldman and Crandall, 2007) or adopting psychiatric treatments, and the psychologist himself. Goffman (1963) defines stigma as “the phenomenon whereby an individual with an attribute which is deeply discredited by his/her society is rejected as a result of the attribute” (p. 21). The stigma is distinguished into public stigma and self-stigma. Both dimensions can potentially express themselves through three different forms: stereotypes, prejudice, and discrimination. As Larson and Corrigan (2010) point out, stereotypes in public stigma refer to societal notions about groups of people, used to organize knowledge. Prejudice indicates adherence to and consensus on the same knowledge associated with negative emotions such as fear, anger, and repulsion. The two forms of public stigma can coexist but stereotypes can also arise regardless of prejudice. Finally, discrimination implies a transition from thoughts and feelings to action.^7^ In the case of the psychologist, an example could be the following: “The psychologist is a fortune teller, I do not address to him”. According to Larson and Corrigan (2010), the patient/the psychologist within self-stigma can be aware of what society thinks about the category (stereotypes), adhere to this knowledge, and develop negative emotions (prejudice; e.g., hating oneself) with a practical impact on their own everyday lives (feelings of discrimination; e.g., Würth et al., 2018). Stigma can be moral or physical, as in self-injuries (Long, 2018). Stigma can have a significant impact on different phases of psychotherapy. It may prevent people from acknowledging that they are struggling with their own feelings and may cause the person to ignore messages from the external (e.g., family) and internal (e.g., panic attack) environments. It may prevent people from recognizing that they need the help of a specialist to the point that they delay or do not undertake the search for a psychotherapist; people may experience low self-esteem or shame for themselves. Since the person does not enter into psychotherapy as a tabula rasa, stigma also acts in psychotherapy in both group and individual treatments by affecting, for example, working alliances (Kendra et al., 2014). In this regard, the self-stigma of mental illness differs and is independent of the self-stigma of seeking psychological help (Tucker et al., 2013). As described by Tucker et al. (2013), a person might seek outpatient counseling or psychotherapy without recognizing themselves as a person in distress. Conversely, another person might recognize himself as a person with a mental illness (and not judge himself for it), but have stereotypes about the therapy and the psychologist. Stigma, and in particular stereotypes, can also refer to the figure of the psychologist and healthcare professionals.^8^ Not all individuals with psychological difficulties decide to consult a psychologist: On the one hand, positive past experiences with psychological services increase the chance of consulting a psychologist (Pfeiffer and In-Albon, 2022); on the other hand, some stereotypes make this event less probable. For example, some adolescents may avoid consulting a school psychologist because they feel that the psychologist might judge them to the point of not being able to help them or because they think that a school psychologist might be useful only to address difficulties closely connected to the school context (Cornoldi and Molinari, 2019). As a result, the individual has to manage dialectical positions concerning the motivations to access the psychological path and the motivations to avoid it (Owen et al., 2013). At the same time, some stereotypes can be explored in therapy by the patient and by the psychologist himself, giving them an active role in this process (Heijnders and Van Der Meij, 2006). Stigma is structurally complex and emanates from different social contexts (e.g., our own ethnic population and institutions). Distinct framings arise from different contextual domains that somehow are connected to the individual. An important distinction needs to be made between these contexts as some of them are related to society (e.g., general population), while others concern the own private social network (e.g., family) with a different impact on the patient and the psychologist (Vogel et al., 2009).

The aim of the present article was to identify the implicit contextual premises that shape particular framings around which the image of the psychologist and the patient are socially and culturally constructed in different contextual domains.

## 3. Methodology

### 3.1. Data collection

We refer to three main sources of data (Table 1): (1) a psychology online forum; (2) different university websites; and (3) an online survey filled out by a heterogeneous sample of citizens.

**Table 1:** Sources and types of data.

**Source of data**	**Types of data**
Psychology online forum	50 posts
University websites	10 sections “professional profiles”
Anonymous online survey	112 answers

We referred to three different contextual domains (our sources of data) since it is only in real-life domains that these types of phenomena can be observed.

We have consulted one Italian informal forum of psychology^9^ which has been selected because it is active for over 15 years. It is one of the main forums about psychology in Italy as it presents different sections, one of which is a free space where lay people can propose a topic of discussion and people can support each other by sharing experiences or answering questions. The forum has been consulted in July 2022 and the latest 50 posts pointing at the social images of the psychologist and of the patient have been considered. The posts have been selected by entering the keyword “psychologist” in the search box and checking the presence of any reference to the person who consults a psychologist. We have copied the posts taken from the forum and pasted them on an *ad hoc* document enabling us to analyze them.

As a second investigation domain, we have consulted 10 Italian university websites for Bachelor's and Master's degrees in psychology: in particular, we have paid attention to the section called “professional profiles”, choosing the more popular ones in terms of those more attended by the students' population. Every university website has such a section presenting the profession of a psychologist and its roles. In order to build the most possible representative sample, we have selected a variety of psychology departments that are different in terms of theoretical framework (e.g., cognitive, psychodynamic, and systemic relational), as well as for their geographical locations (universities from South, Center, and North of Italy). In a similar way, as for the informal forum, we have copied and pasted in an *ad hoc* document the concerned sections, to have the possibility to analyze it. The 10 university websites were consulted in July 2022.

The third context of investigation is represented by informal public opinions of a heterogeneous sample of citizens gained through an anonymous online survey in which we asked people two questions: “What comes to your mind when you think of a psychologist?” and “When is it advisable to contact a psychologist?” The first general question was chosen to set the context and to introduce people to the second – more specific – question, in order to elicit information on possible lay people's prejudices and stereotypes about the profession and about the patient. Since we were not interested in evaluating the correctness of the answers nor drawing up normative profiles, we have proposed the above-mentioned two open questions rather than referring to a validated questionnaire. The survey has been launched through Google Forms^10^ and submitted via social media or e-mail to a group of 72 people representing a variety of social profiles in order to maximize the possibility of having a heterogeneous group of respondents.^11^ The invitation to fill out the survey has remained active for 4 months, from April 2022 to July 2022. No data about respondents are reported here, as the survey was anonymous. The answers to the questions did not entail space limits: participants were free to express themselves without any restrictions. We obtained 55 answers to the first question and 57 to the second question (112 in total) which were then copied and pasted in an *ad hoc* document for the analyses.

### 3.2. Analytical approach

We stage our research in the context of the polylogical argumentation (Lewiński and Aakhus, 2014)^12^ which allows us to put into relation different points of view around the same topic (in our case, the representations of the psychologist and the patient). To analyze our data, we have used the pragma-dialectics (van Eemeren and Grootendorst, 1984, 2004) to define the issue and to identify the standpoint and the arguments supporting it. Concerning the missing link represented by implicit premises, we have used the Argumentum Model of Topics (AMT, Rigotti and Greco, 2019) for studying the inference and to reconstruct the implicit premises. After these steps, we have reconstructed the distinct framings at stake in the different contextual domains (related to our sources of data: psychology online forums, university websites platforms, and the online survey). We have analyzed the framings at two levels: at a micro level, by looking at words and expressions according to what has been done by Fillmore (1976); and at a macro level, according to what has been done by Goffman (1974). We recognize that we were looking at the data through the lenses of our premises and prejudices^13^ (Cecchin et al., 1997) that guided us in understanding others' premises and prejudices, in line with the second cybernetic (Maturana and Varela, 1980).

## 4. Findings

The performed analyses show as a main result that implicit contextual premises have huge power in constructing stigmatizing framings and, more in detail, in highlighting the stereotypes that rotate around the images of the psychologist and of the patient. We have observed that the more the contextual domain is defined (as in the case of the university website), the more group premises dominate over individual ones; on the contrary, in informal heterogenous contexts (as in the case of the online forums and the survey), individual premises are prominent and consequently more heterogeneous. Indeed, on the basis of the analyses of the university websites, a homogeneous picture of the premises on the patient, on the psychologist, and of whom are consulting a psychologist arises. This can be considered a normative view of the psychologist. On the contrary, these premises are heterogeneous in the answers elicited from the psychology informal forums and the survey.^14^ In more detail, university websites support the figure of the psychologist as a professional who promotes psychological wellbeing in various fields. Data from the anonymous online survey present different types of stereotypes and prejudices: The most frequent concerns the idea of psychology centered on a curative approach and wellbeing as the absence of symptoms; less frequent, but still present, is the idea of the psychologist as a psychoanalyst (e.g., the presence of a coach and the idea of psychologist as the one who makes people speaking for a long time); in some excerpts, the psychologist is considered less important and less useful than the psychiatrist. Although the term “psychologist” has been adopted in the survey, most of the participants refer to the psychotherapist. In the online forum, we find similar categories of stereotypes and prejudices as presented in the survey. However, sentences are accompanied by more qualifying adjectives. Disgust emotions could be addressed to the psychologist, the patient (in both other-directed and self-directed forms), and the people surrounding the patient. To highlight the role of implicit premises in stigmatizing framings, we will present four examples, grouping the less representative items for each different contextual domain. We present these excerpts as they describe less visible forms of stigma. Our first example is taken from a university website (Section 4.1), the second one (Section 4.2) is related to the psychology online forum, and the last one (Sections 4.3 and 4.4) concerns the anonymous online survey.

### 4.1. Excerpt 1: psychology beyond psychic distress

The following excerpt is taken from the website of the University of Turin (Italy) on the presentation page of the Bachelor of Arts - BA in “Psychological Sciences and Techniques”.^15^ More specifically, our analysis focuses on the section about the occupational profiles of future graduate students. In this section, the main tasks and opportunities for graduate students are presented referring to current Italian legislation (DPR 328/01) and the Order of Psychologists (psychologists can enroll in this order after having passed a formal examination qualifying them to the profession).

In Excerpt 1, we analyze the part in which the main professional opportunities are presented with a focus on what the psychologist will practically do and the contexts in which they will operate.

According to the pragma-dialectics approach, the following elements can be identified (Table 2):

*Issue*: Can bachelor graduate students carry out some professional activities?
*Standpoint*: The graduate students will be able to carry out professional activities in public as well as private structures, in educational institutions as well as in third-sector organizations.
*Argument*: Because the course prepares to carry out professional activities as collaborators of psychologists enrolled in the Order in different organizational and research contexts.

**Table 2:** Excerpt taken from a university website.

**Original verbatim**	**English translation**
Il Corso di Laurea prepara a svolgere attività professionali in qualità di collaboratore dello psicologo iscritto all'Albo A, nell'ambito di diversi contesti organizzativi e di ricerca. Di conseguenza, i laureati della classe potranno svolgere attività professionali in strutture pubbliche e private, nelle istituzioni educative, nelle imprese e nelle organizzazioni del terzo settore.	The degree course prepares graduate students to carry out professional activities as collaborators of the A-listed psychologists in various organizational and research contexts. Consequently, graduate students of the concerned class will be able to carry out professional activities in public and private facilities, educational institutions, companies, and third-sector organizations.

In the example, the issue at stake for the argumentation can be identified as follows: “Can bachelor graduate students carry out some activities?” The present issue is implicit in the quotation and can be made explicit starting from the initial focus of the sentence that highlights the professional activities that bachelor graduate students can enact. The issue pertains to the domain of possibilities and is placed out publicly. It is strictly dependent on the type of website and section: this issue is in line with the university context and strongly interacts with the meta-message that advises students to enroll in the Bachelor's degree in psychology. The adjective “professional” specifies and delimits the borders of activities that the psychologist belonging to a specific community of professionals can enact. In our analytical reconstruction, the standpoint is the following: “The graduate students will be able to carry out professional activities in public as well as private structures, in educational institutions as well as in third-sector organizations”. The university puts forth a standpoint that fosters a positive image of the psychologist as a professional that not only cures psychic distress but rather helps to increase psychological wellbeing and to prevent psychological distress. The argument supporting this standpoint is the following: “Because the course prepares to carry out professional activities as collaborator of the psychologists enrolled to the Order in different organizational and research contexts”. This argument invites the audience to reflect on the fact that being a professional requires attending an academic path, therefore implicitly arguing that a psychologist is a professional.

**Figure 1:**
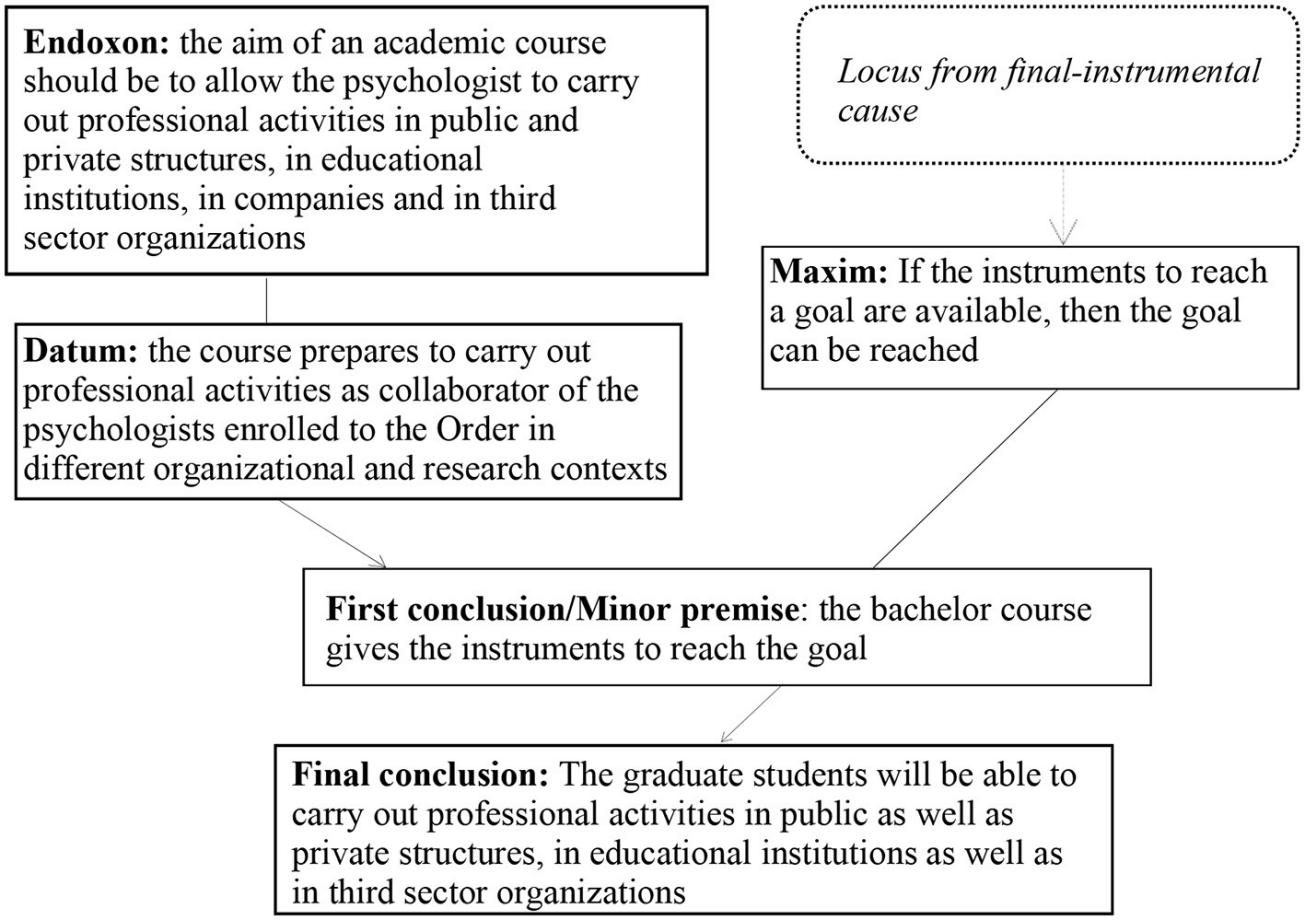
Argumentative structure of the argumentation in Excerpt 1.

Figure 1 presents the argumentative structure of the argumentation.

The AMT enables us to make an inferential analysis and to consider the reasoning passages that make it possible to draw a conclusion on the basis of a given premise: in particular, this method enables us to shed light on the implicit premises and to make them explicit. In our analysis of framings, the endoxon is a crucial element because it coincides with the cultural ingredient of the reasoning that is supposed to be shared and accepted by a given community. This endoxon is fundamental to the image of professionalism that the university wants to convey. The endoxon is not simply an implicit element in the discourse, but is a premise in the reasoning, i.e., without this component, the reasoning does not allow the conclusion to be reached. In our example, the endoxon “The aim of an academic course should be to allow the psychologist to carry out professional activities in public and private structures, in educational institutions, in companies and in third-sector organizations” is not only specifically accepted by the university but it is also an element that the concerned university considers to be universally shared and without which it is not possible to conclude that “The graduate students will be able to carry out professional activities in public as well as private structures, in educational institutions as well as in third-sector organizations”. Through the implicit endoxon, the university supports the figure of the psychologist as a professional who promotes psychological wellbeing in various fields (e.g., Ryff and Keyes, 1995). Finally, this principle is not only adopted by the concerned university but it is also recognized by the university itself as fundamentally accepted by every academic institution.

### 4.2. Excerpt 2: “you are a psychologist, you should tell me what to do”

The following excerpt is taken from informal public opinions of a heterogeneous sample of citizens. In this section, we analyze an answer to the question “What comes to your mind when you think of a psychologist?” of the proposed anonymous online survey. We decided to focus on this specific example because, in our opinion, it is significant of common people's premises concerning the psychologist and the psychological intervention.

In Excerpt 2, we analyze a sentence in which a person firmly and concisely offers an opinion on who is a psychologist (Table 3).

**Table 3:** Excerpt from the online survey.

**Original verbatim**	**English translation**
Un professionista che dia una risposta ai tuoi mille interrogativi.	A professional who should give me an answer to my endless questions.

According to the pragma-dialectics approach, the following elements can be highlighted:

*Issue:* What comes to your mind when you think of a psychologist?
*Standpoint*: The psychologist is the one who should give me an answer to my endless questions.
*Argument:* Because the psychologist is a professional.

In our example, the issue at stake for the argumentation coincides with the question proposed in the survey: “What comes to your mind when you think of a psychologist?” As a result, the present issue is made explicit and does not need to be reconstructed. In our analytical reconstruction, the standpoint is the following: “The psychologist is the one who should give me an answer to my endless questions”. Starting from the standpoint, we can reconstruct the implicit issue opened by the participant: “Does it exist a right answer to my endless questions?” Indeed, in the standpoint, the participant adopts the Italian formulation “una risposta” that could be interpreted as “one answer” or “an answer”. However, the core of the matter does not change since the patient expects that the psychologist reduces the field of possibilities opened up in the participant's mind. The paradox consists in the fact that, as psychologists, we have the ethical duty to do the opposite, and to broaden the field of possibilities in line with Von Foerster's ethical imperative: “act always so as to increase the number of choices” which the father of the second order cybernetics proposed in his essay (von Foerster, 1984).

**Figure 2:**
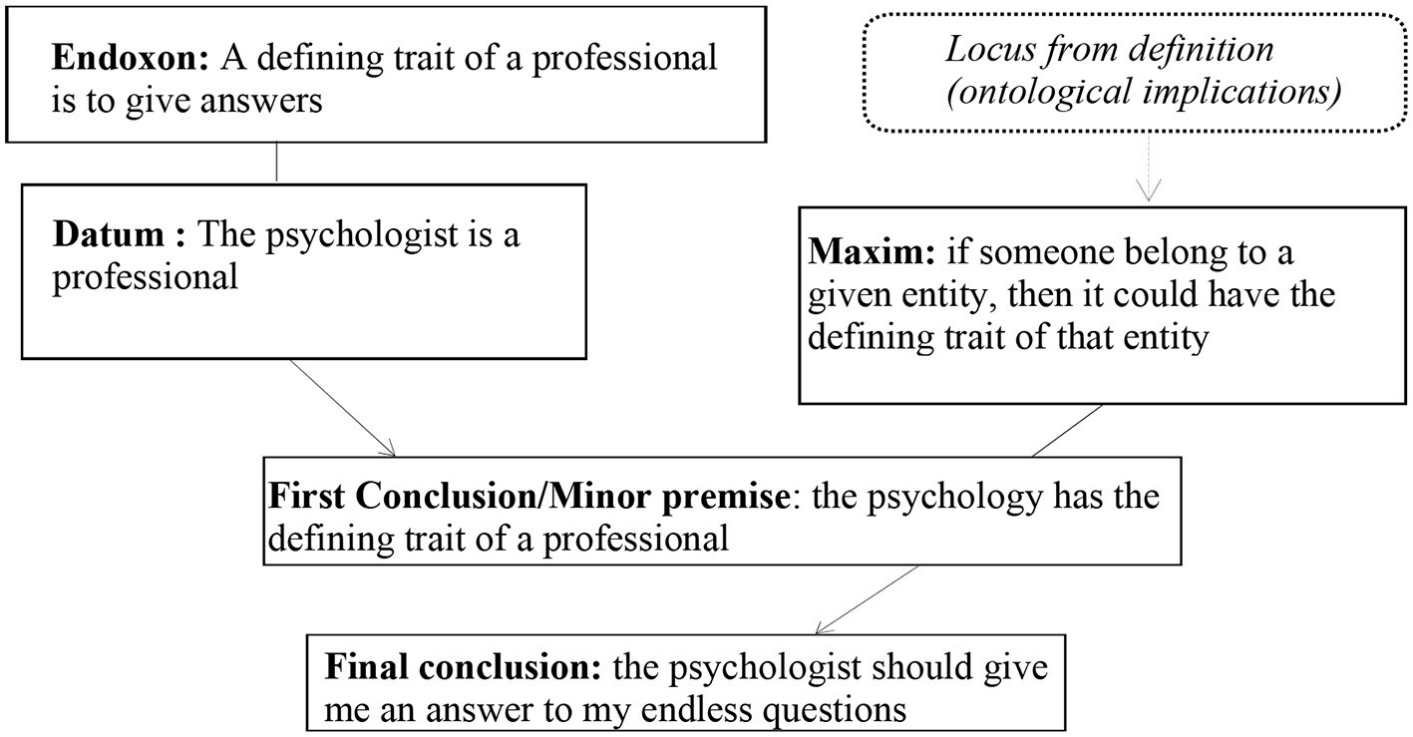
Argumentative structure of the argumentation in Excerpt 2.

In what follows, we show the analysis of the inferential configuration of the participant's argumentation (Figure 2).

The inferential reasoning of the argumentation describes a definitional procedure that derives from the objectives of a professional, i.e., the definition of the psychologist is built on the goal of a professional.^16^ The inference connecting the argument and the standpoint is embodied in the locus from definition (ontological implication). As stated by Schär (2017, pp. 179–180), “the locus designates the relationship between the nature of an entity and the implications of this nature, namely the goal this entity has been designed for. An example that highlights the relation between ontology and deontology would be: if you are a politician, you need to be accountable”. This example highly reminds of our case, in which the locus identifies the relationship between the essence of the ontology of the psychologist and its deontological implications, namely “telling the patient what to do” (i.e., “the psychologist should give me an answer to my endless questions”) in the final conclusion. Such a stereotype has the negative consequence of removing responsibility from the patient, who at the same time loses a high degree of freedom and agency in his life. On the contrary, in psychotherapy, the patient's disclosure has a real healing power and has *a construens* function: the patient selects given pieces of information that are then deployed by the therapist to reframe the patient's narration, so that the patient can re-read reality in a more useful way for them in a given moment (Luciani and Convertini, 2023).

### 4.3. Excerpt 3: “the patient as an abandoned child”

The following example is taken from the informal public opinions of a heterogeneous sample of citizens, as in Section 4.1. In this section, we analyze an answer to the question: “When is it advisable to contact a psychologist?” We present this specific excerpt because it sheds light on a very subtle stereotype that involves both the patient and the people around them. In particular, in Excerpt 3, we analyze a sentence in which a person expresses an opinion on his view of who a patient is (Table 4).

**Table 4:** Excerpt from the online survey.

**Original verbatim**	**English translation**
Quando non si ha nessuna figura vicina che sia disponibile ad aiutarti e/o che non abbia gli strumenti teorici per farlo.	When you do not have any person close to you who is available to help you and/or who does not have the theoretical tools to do that.

The reconstruction of the argumentation is staged within the framework of pragma-dialectic:

Issue: When is it advisable to contact a psychologist?
Standpoint: When people close to you cannot help you.
Argument 1: Because they are not available.
Argument 2: Because they do not have the theoretical tools to do that.

**Figure 3:**
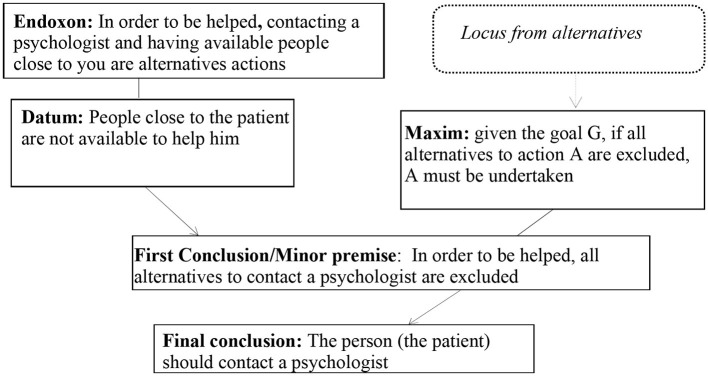
Argumentative structure of the argumentation in Excerpt 3.

According to our reconstruction, the issue is the following: “When is it advisable to contact a psychologist?” The implicit standpoint proposed by the participant to answer the issue is the following: “When people close to you cannot help you”. The participant explicitly expresses two arguments that support the standpoint, namely: “Because they are not available” and “Because they do not have the theoretical tools to do that”. These arguments can be considered independent from each other since they appeal to distinct characteristics of the person nearby. Indeed, a person could have the theoretical instruments to help you, but they could be unavailable to help you and, vice versa, a person being available to help you might not have the theoretical tools to do that. As we identified the same logical principles for both Argument 1 and Argument 2, in what follows, we only show the analysis of the inferential configuration of the participant's argumentation Argument 1 (Figure 3):

The first stereotype that emerges from our reconstruction is that the patient is a person who is completely alone in getting help. In turn, this stereotype is bound with the ancestral stereotype of the psychologist conceived as someone who keeps isolated members company. On the contrary, a person may be isolated (since they may be part of a familiar as well as social system that leaves them at the margins), but not alone since they may be surrounded by destructive relationships. Furthermore, the psychologist is conceived almost as a friend that should answer to the patients' familiar and social needs. The premises connecting the first argument (“Because they are not available”) with the standpoint (“When people close to you cannot help you”) signal that the person who is close to that patient does not help the patient because they do not want or that they cannot. In short, the pragmatic effect is that the patient does not receive help from the person who is close to them. A prejudice emerges that the familiar and social contexts around the patient are poor in relational resources. The premises connecting the second argument (“Because they do not have the theoretical tools to do that”) with the standpoint (“When people close to you cannot help you”) signal that even in the hypothesis that the person who is close to the patient is available to help them, they are unable to do that. Theoretical instruments are not the unique and most important tools in a psychotherapy path. Moreover, the network of a patient could not have theoretical instruments to help the patient, but rather could have relational as well as human resources able to help them (e.g., Kucharewicz and Wieteska, 2019).

### 4.4. Excerpt 4: “yours are not problems!”

The following excerpt is taken from a psychology website in which articles written by experts as well as an online forum are included. In the online forum, private users can publish questions about different topics (such as naïve advice on psychological issues and on the therapeutic path to choose) and they can receive answers from both experts and lay people.

In Excerpt 4, we present a dialogical exchange involving two users. In particular, we analyze a question proposed by a user and the answer advanced by another one. In this excerpt, the participants do not explicitly discuss the figure of the “patient”. However, we present this discussion because they reflect on patient' feelings and to some extent on the meaning of being a patient (Table 5):

**Table 5:** Extract from informal forums of a psychology website.

User 1	I am a lonely person and I seek solitude. Although I feel an initial relief, it turns into sadness afterward. Why do I keep doing it then?
User 2	The youth of today is confused, we are unable to find an identity. In the past, human beings experienced similar feelings, but they didn't get lost in them and didn't see them as a problem. Today, we find everywhere problems that need to be solved. Loneliness is not a problem. Yours are not problems, they are normal things. If you go to consult a psychologist, they will find you a problem, but I would like to know what normality is.

The reconstruction of this argumentation is staged within the framework of pragma-dialectics:

Issue: Should I ask myself these questions?
Standpoint 1: (no)
User 2 Arg 1: Because feelings of being confused and failure to find an identity are not a problem but normal things
User 2 Arg 1.1: Because in the past human beings didn't see them as a problem
User 2 Arg 2: Because we find problems everywhere that need to be solved
User 2 Arg 3: Loneliness is not a problem
Issue: Should I consult a psychologist?
Standpoint: (no)
User 2 Arg 1: Because he will find you a problem
User 2 Arg 2: Because the normality does not exist.

A question arises from User 1: “Why do I keep doing it then?” However, User 2 shifted the issue. According to our reconstruction, the implicit issue that can be reconstructed starting from the sentence of User 2 is the following: “Should I ask myself these questions?” The implicit standpoint proposed by the participant to answer this issue is “No”. The participant explicitly expresses several arguments in a combination of different argumentative structures (van Eemeren and Grootendorst, 1992). The arguments supporting the standpoint are: “Because feelings of being confused and failure to find an identity are not a problem but^17^ normal things”. This argument is, in turn, supported by another argument (Arg 1.1): “Because in the past human beings didn't see them as a problem”. In this subordinative structure, argument 1.1 strengthens the standpoint. User 2 presents two additional arguments: “Because we find problems everywhere that need to be solved”, and “Loneliness is not a problem”.

With reference to the implicit issue “Should I consult a psychologist?”, the implicit standpoint of User 2 is “yes”. The participant explicitly expresses two arguments that support the standpoint, namely “Because he will find you a problem”, and “Because the normality does not exist”.

The first stereotype that emerges from our reconstruction is that being a very reflective person coincides with being a problematic person. On the contrary, we argue that being a reflective person is the expression of being a person with very high meta-reflective functions and a person capable of looking into reality with a very critical glance. The process that underways the exchange in Excerpt 4 can be seen as a form of “emotional invalidation” (perceiving one's own emotions as trivialized or ignored by the other).^18^

Furthermore, a second stereotype that emerges from our reconstruction is that a psychological issue exists only if it is recognized by another person and in particular a person who is an expert in psychological health, such as a psychologist. On the contrary, a person consulting a psychologist is already doing a step forward in the treatment process. Indeed, if the patient has not a first insight toward the recognition of a psychological issue, they would not consult a psychologist.

## 5. Discussion and openings

The state of the arts concerning stigma in psychotherapy has highlighted that stereotypes, prejudices, and discrimination refer to psychologists well as to patients. This mechanism is at the very core of avoiding and delaying psychotherapeutic interventions. Furthermore, it strongly influences the session's flow. It also affects people's private and professional lives, as well as their mental health.

To move a step forward within this frame, we have undertaken a different theoretical and methodological approach. From a theoretical point of view, we consider that the representations of the psychologist and of the patient are socially co-constructed. A confusion around as to whom “psychologists” and “patients” are may underlines (implicit) stereotypes and, broadly speaking, stigmas related to mental health (WHO, 2022). Therefore, we have investigated argumentations proposed in different contextual domains to address the issue of who the patient and the psychologist are. From a methodological point of view, we have focused on implicit discourses by making explicit the missing link explaining the passage from implicit premises to conclusions, and its interweaving with specific framings.

In our view, contextual premises represent the very core of stereotypes' (and stigmas') construction. Through four excerpts, we have been able to show the following aspects: (a) university endorses a positive image of the psychologist, as a professional who follows a very specific training in order to be competent to increase personal growth and life purposes; (b) lay people who answered to our survey offer a representation of the psychologist as an expert person who gives advices and takes responsibilities in the place of the patient, thus restoring an image of the patient as a person without agency; (c) lay people advance a representation of the patient as a person who is completely alone and, in some way, isolated from social reality (the familiar and social contexts around the patient are poor of relational resources); (d) lay people's opinions from online forums highlight that being a very reflective person coincides with being a problematic person and that a psychological issue exists only if it is recognized by another person and in particular by a person who is expert of psychological health.

## 6. Conclusion

In conclusion, we believe that contextual implicit premises are fundamental in shedding light on how stereotypes (and stigma) are built according to how people see the world and themselves. It is the perfect idea (Cecchin and Apolloni, 2003) of how the world should be that constructs prisons around people's lives: “As therapists we should open up a space of freedom from tangles of maladaptive habits” (Luciani, 2021, p. 130).

As stated by Bakhtin (1986), “contextual meaning is potentially infinite, but it can only be actualized when accompanied by another (other's) meaning, if only by a question in the inner speech of the one who understands …There can be no ‘contextual meaning in and of itself' –it exists only for another contextual meaning, that is, it exists only in conjunction with it” (p. 145). In psychotherapeutic terms, this means that the heart of therapy (and therefore of change) can be found at the very core of the dialogue between the premises of the patient and those of the therapist (Cecchin et al., 1997): for instance, if a patient strongly uses premises relying on authority judgment (“I have done x because my mum/my boss/my professor has said that…”), the therapist can counter-argue with premises referring to other types of meaning (“I have done x because I felt that it made me relax”).

At a more theoretical level, we can conclude that perfect ideas arise when the Self does not dialogue with the other and more particularly when our premises do not dialogue with each other and with the others' premises. Therefore, it is only by putting our premises in connection among each other and with the others' premises (and especially individual premises with institutional premises) that we can fight mental health stigma.

Our study has highlighted the importance of transforming implicit stereotypes into a visible object of study, for disentangling elements around the social images of the psychologist and of the patient in communication. Starting from this path, we can change subjacent contextual premises at the very core of stigmas and prototypical world images only by making explicit the implicit premises. Our aim was to reconstruct the prototypical social images of the psychologist and of the patient, as it could be dangerous when it prevents people from asking for psychological help and taking care of their psychological wellbeing. As people intending to promote taking care of human wellbeing, we should fight this reductionist view that is still dominant in our society. In this regard, education and school are also crucial because “ensuring rights-based approaches to mental health should be incorporated into human rights education programs in schools as well as feature as compulsory core components of tertiary and vocational education and specialized training for health and legal professionals, the police and judiciary to combat discrimination and negative stereotypes” (WHO, 2022, p. 117). Our study has shown that further research is needed in the field of mental health in order to consider the following open questions: How can we intervene in fighting the mental health stigma? What type of training and methodologies can be useful? We strongly believe that our combination of theoretical and methodological approaches could be beneficial for future investigations in the domain of argumentation and communication in psychology. This will allow us to better consider the role of implicit premises that are vehiculated in the construction of social representations of actors interacting in professional contexts, such as psychologists and patients.

## Data availability statement

The raw data supporting the conclusions of this article will be made available by the authors, on request.

## Ethics statement

The participants provided their written informed consent to participate in this study.

## Author contributions

All authors listed have made a substantial, direct, and intellectual contribution to the work and approved it for publication.

## Footnotes

^1^See https://europsy.eu/_webdata/europsy_regulations_december_2021_virtual_ga.pdf (last accessed 10 July 2023).

^2^The original verbatim is: “La professione di Psicologo comprende l'uso degli strumenti conoscitivi e di intervento per la prevenzione, la diagnosi, le attività di abilitazione-riabilitazione e di sostegno in ambito psicologico rivolte alla persona, al gruppo, agli organismi sociali e alle comunità. Comprende altresì le attività di sperimentazione, ricerca e didattica in tale ambito”. For more details, see https://www.gazzettaufficiale.it/eli/id/1989/02/24/089G0090/sg (last accessed 5 May 2023).

^3^For more details, see https://dictionary.apa.org/psychologist (last accessed 4 July 2023).

^4^We will adopt the term “patient” in order to avoid semantic ambiguities.

^5^“There is an imperative to transform the way mental health c-are and support are conceived and provided (...) there is growing recognition that mental health and well-being are intimately linked to one's social, economic, and physical environment” (WHO, 2022, p. 16).

^6^Our translation. The original Italian quotation is the following: “derivati di azioni e comportamenti pragmatici, finalizzati a gestire il presente, che in seguito all'evidenza della loro utilità ed efficacia tendono a venire strutturati in termini di verità assoluta, atemporale e astorica”.

^7^For an extended overview of discrimination in intellectual disability (ID), see the works of Ebersold (e.g., Ebersold and Armagnague-Roucher, 2017; Ebersold, 2022).

^8^e.g., Buttigieg et al. (2018), Zawawi and Al-Rashed (2020), Kwaghe et al. (2021), and Najjar et al. (2022).

^9^https://www.psiconline.it/forum/ (last accessed 5 May 2023).

^10^https://www.google.com/intl/it/forms/about/ (last access, 5 May 2023).

^11^Due to the fact that we consider stigma as a socially widespread phenomenon (Sanhori et al., 2019), we have not collected socio-demographical data about the sample.

^12^Aakhus and Lewiński (2017) define “argumentative polylogue not simply as a discussion between multiple participants, but rather multiple different argumentative parties defending their distinct positions” (p. 181). For more details on the application of polylogical argumentation to the study of multiple voice actors, see Greco and De Cock (2021) and Greco (2023).

^13^Cecchin used the term prejudices in order to refer to premises so that he could distinguish himself from the logicians of the time.

^14^In order to give a picture of the examples that are present in our corpora, we might refer to some approximate figures. We analyzed 172 excerpts in our study. Around 40% of these examples (*N* = 69) are, in our interpretation, cases in which stereotypes and prejudices emerge. Our calculations are dependent on our interpretation as analysts of argumentation and related to the lenses of our premises and prejudices (Cecchin et al., 1997). Although these figures might be subject to debate, in this study, our objective is to emphasize the role of implicit premises in stigma, without pretending any statistical form of generalization.

^15^The original Italian course's name is ≪Scienze e Tecniche Psicologiche≫.

^16^As stated by Greco et al. (2018)., “The ontological implication on which this argument is built derives from this kind of functional definition” (p. 452).

^17^The adversative connective “but” helps to signal the argumentation (Rocci et al., 2020).

^18^In clinical psychology, “emotional invalidation” has a key role in disorganized mother-child attachment patterns (e.g., Liotti, 2001, 2004).
